# The structural effects of indel polymorphisms outside the binding site on RNA-protein interactions are shaped by selection

**DOI:** 10.1371/journal.pcbi.1013604

**Published:** 2025-10-21

**Authors:** Carlos Owusu-Ansah, Elan Shatoff, Ralf Bundschuh

**Affiliations:** 1 Interdisciplinary Biophysics Graduate Program, The Ohio State University, Columbus, Ohio, United States of America; 2 Department of Physics, The Ohio State University, Columbus, Ohio, United States of America; 3 Center for RNA Biology, The Ohio State University, Columbus, Ohio, United States of America; 4 Department of Chemistry & Biochemistry, The Ohio State University, Columbus, Ohio, United States of America; 5 Division of Hematology, The Ohio State University, Columbus, Ohio, United States of America; University of Missouri, UNITED STATES OF AMERICA

## Abstract

Genomic variants influence phenotypes and organismal fitness, with their effects shaped by genomic context. In 3’ untranslated regions, variants can alter phenotypes by influencing RNA-protein binding and subsequent post-transcriptional gene regulation. Here, we investigate how indel variants impact RNA-protein interactions from outside the binding site, through changes in RNA secondary structure. Our findings reveal that indels can significantly affect protein binding affinities over distances spanning tens of nucleotides, with longer indels exerting greater effects until saturation. Crucially, we find evidence that this effect is constrained by purifying selection. Naturally observed indel polymorphisms cause smaller changes in binding affinity than synthetic indels, and this signal of selection is more pronounced near HuR binding sites. We also find that the sequence context of HuR binding sites shows greater resilience to indel mutations compared to randomly selected sites. These results demonstrate that indel polymorphisms outside the binding site can modulate RNA-protein interactions through structural effects, and that purifying selection acts to filter out variants that disrupt critical interactions.

## Author summary

Genetic differences between individuals can influence how genes are regulated, leading to changes in health outcomes. While variants called indels are known to influence regulatory proteins by altering their direct binding sites, our study investigates how these variants affect RNA-protein interactions from outside the binding site by altering the structure of RNA molecules. We discovered that indels far from protein binding sites can significantly weaken or strengthen binding, with larger indels having a bigger impact. Interestingly, our analysis shows that natural selection works to minimize such disruptions: indel polymorphisms that greatly disturb RNA-protein binding are less likely to persist in populations. Moreover, protein binding sites near key regulatory regions, such as those bound by the HuR protein, are more resilient to the effects of indels. These findings highlight how genetic changes can influence gene regulation indirectly and show that the genome is shaped to protect critical regulatory mechanisms from such disruptions. This work sheds light on the interplay between genetic variation, RNA structure, and gene regulation, advancing our understanding of how genetic changes impact health and evolution.

## Introduction

Genomes within a species exhibit variation between individuals. For example, the dbSNP database catalogues over 1.1 billion variants in the human genome [1]. Of these, approximately one billion are single nucleotide polymorphisms (SNPs) while the remainder are primarily insertions or deletions (indels). While not every variant has phenotypic consequences, together they drive the phenotypic differences observed between individuals. The mechanism by which variants affect phenotypes depend on their genomic locations. Variants in coding regions of protein coding genes can directly change the amino acid sequence of the corresponding protein, those in promoters or enhancers can influence transcription rates, and those in introns can affect splicing and/or other RNA processing steps.

Here, we focus on how genetic variants induce phenotypic changes by altering the affinity of a messenger RNA (mRNA) for regulatory RNA-binding proteins (RBPs). The 3’ untranslated regions (3’UTRs) of mRNAs are hotspots for RBP binding, which in turn regulates mRNA localization, stability, and translation [2]. The binding of an RBP depends not just on the primary sequence of its motif, but also on the RNA’s secondary structure, which can expose or conceal binding sites. This structural context is a determinant of RBP binding specificity and affinity [3].

A variant within an RBP binding site is likely to affect binding. However, because structure is paramount, changes located far from the binding site can also have profound effects [4–6]. This is well-established for single nucleotide polymorphisms where so-called "RiboSNitches" can trigger conformational changes in the RNA that distally modulate RBP-mediated processes [7]. Yet, while the concept of structure-mediated distal regulation is known, the impact of insertion and deletion polymorphisms (indels) has not been systematically quantified. Furthermore, while it is established that indels in functional regions are broadly subject to purifying selection [8], it is unknown whether this selection pressure specifically targets allosteric, structure-mediated effects of indels on RBP-RNA interactions.

To investigate these questions, we use the RNA-binding protein HuR (Human Antigen R) as a model system. Encoded by the ELAVL1 gene, HuR is ubiquitously expressed and plays a critical role in post-transcriptional gene regulation [9]. It binds to AU-rich elements (AREs) and U-rich sequences found in the 3’ untranslated regions (3’UTRs) of target messenger RNAs (mRNAs), such as those for cytokines, growth factors, and oncogenes [10]. Binding by HuR stabilizes these mRNAs, leading to increased protein production [11,12]. Binding by HuR is mediated by its well-characterized RNA Recognition Motif (RRM) domains, which facilitate high-affinity interactions with target sequences [13,14]. Given its fundamental role in cellular processes, frequent dysregulation in diseases like cancer, and its well-characterized RNA-binding properties, HuR is an ideal model for investigating how RNA-protein interactions are modulated by genetic variants [12].

Previously, we demonstrated computationally that single nucleotide changes can significantly alter HuR binding affinity at distances of up to 50 nucleotides away, and that these effects appear to be under selection [15]. In this study, we extend this work to indel variations to address outstanding questions about their functional impact. We systematically explore how the at-a-distance effect of an indel on RBP binding affinity depends on its size, and test for the presence of selective pressure against distal, indel-induced disruptions of RBP-RNA interactions.

## Results

### Computational quantification of indel effects on RNA-protein binding.

Our approach, based on previous work [4–6,15,16], models RNA-protein binding as base-pairing constraints that disrupt RNA secondary structure. The change in Gibbs free energy upon protein binding (ΔG) is calculated as the difference between the folding free energy of the RNA with the binding site constrained (made single-stranded) versus that of the unconstrained RNA ($G_{\text{constraint}}-G_{\text{none}}$). This ΔG reflects the energetic cost of making the binding site accessible. Contributions from direct protein-RNA contacts are assumed to be identical when comparing alleles since the binding site sequence itself is unchanged by the indel, and thus cancels out.

To calculate these folding free energies, we used the ViennaRNA package [17]. This software models RNA secondary structure by defining the ensemble of all possible secondary structures that are formed from canonical Watson-Crick and GU “wobble” base pairs and are free of pseudoknots. The thermodynamic stability of each potential structure is then evaluated using an additive nearest-neighbor model, where the total free energy is the sum of experimentally determined energy contributions from individual motifs. For our calculations, we employed the software’s default Turner 2004 energy parameters [18]. Crucially, rather than focusing on a single minimum free energy (MFE) structure, we computed the full partition function over the complete ensemble of possible structures. This approach yields a partition function free energy [19], which provides a more robust and thermodynamically accurate measure of structural stability by accounting for the contributions of all possible conformations [20].

Our analysis focuses on pairs of RNA sequences, termed alleles, that differ by an indel: a longer allele (l-allele) representing the transcript variant with more nucleotides, and a shorter allele (s-allele) representing the variant with fewer nucleotides. The differential binding preference of an RBP for one allele over the other is quantified by $\Delta \Delta G=\Delta G_{\text{l}}-\Delta G_{\text{s}}$. In our model, the effect of the indel on protein binding is indirect. Since the indel is located outside the binding site, its nucleotides do not directly contact the protein; instead, the indel alters the ensemble of possible RNA secondary structures, modifying the energetic cost of exposing the binding site. A positive ΔΔG indicates a stronger binding preference for the s-allele, while a negative ΔΔG indicates a stronger preference for the l-allele.

To investigate the impact of indels and explore signatures of natural selection, we analyzed several categories of indels and sequence contexts:

1. **Naturally occurring human indels:** These are indel polymorphisms sourced from dbSNP [1], representing variations observed in human populations. We analyze these within their native 150-nucleotide human sequence context, centered on the indel. These provide a baseline for the effects of indels that have persisted in the population.
2. **Synthetic indels in random sequences:** To understand the biophysical impact of indels in the absence of any sequence-specific selection pressures inherent to evolved genomes, we generated synthetic indels by computationally deleting segments of varying lengths within 150-nucleotide random RNA sequences.
3. **Synthetic indels in human sequences:** To distinguish between general properties of human sequences and selection acting specifically on indel locations, we also analyzed synthetic indels generated within 150-nucleotide segments randomly extracted from human transcripts.
4. **Binding site context for human sequences:** For analyses involving human transcript sequences, we further distinguish effects measured at arbitrary binding locations versus those measured at or near known RBP (i.e., in our case, HuR) binding sites. This allows assessment of whether selection is more pronounced at functionally relevant positions within human transcripts.

**Fig 1:**
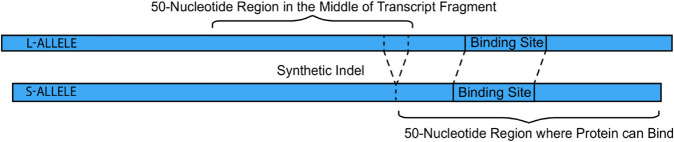
Creating synthetic indels and binding sites. Simulations are performed on a 150-nucleotide RNA fragment (l-allele). A synthetic indel is created by deleting nucleotides of varying length (from 1 up to 50) from the 3’ end of a central 50-nucleotide region (positions 51-100). The indel is thus effectively located at position 100. To measure its impact, all possible 7-nucleotide binding sites in the adjacent 50-nucleotide downstream region (positions 101-150) are systematically tested. The closest a binding site can be to the indel is at position 101; the closest it can be to the end of the fragment is when its final nucleotide is at position 150.

The generation of synthetic indels (types 2 and 3) is illustrated in Fig 1. By comparing the magnitudes of ΔΔG across these categories, we can investigate how natural selection influences the impact of indels on RBP binding.

### Indels can significantly alter RNA-protein binding affinity, with effects that are symmetrically distributed and diminish with distance.

**Fig 2:**
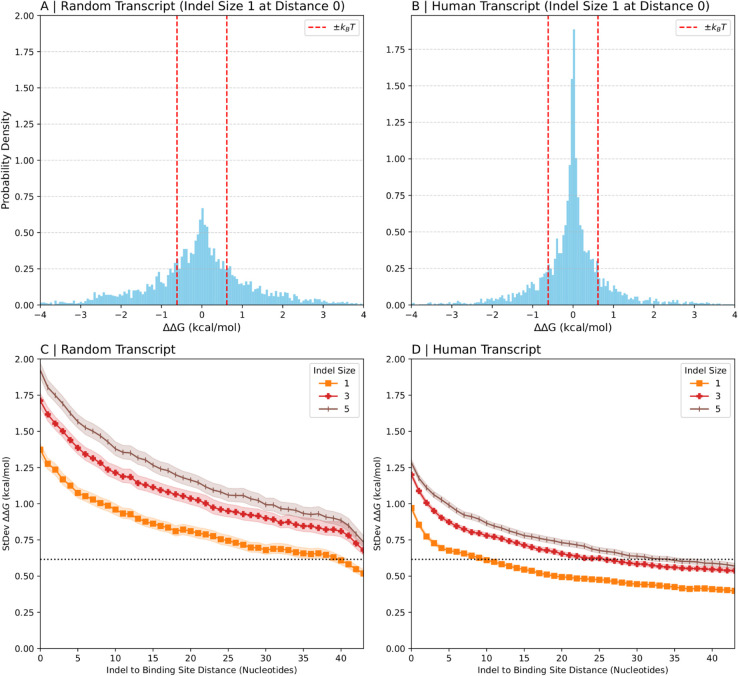
Distribution of binding preferences. (A, B) Histograms of binding preferences for synthetic indels in random transcripts fragments (A) or natural indels in human transcript fragments (B) of length 150nt are shown for indels of size 1 that are 0 nucleotides from the binding site. (C, D) Standard deviation of the binding preference versus distance from the indel for synthetic indels in random transcripts (C) and natural indels in human transcripts (D). Each panel contains three lines which correspond to indels of size 1 (orange), 3 (red) and 5 (brown). Shaded regions represent 95% confidence intervals. The dotted vertical and horizontal lines indicate thermal energy *k*_*B*_*T*.

We first investigated the overall distribution of binding preferences (ΔΔG) induced by indels. A key question is whether indels systematically favor binding to either the longer (l-allele) or shorter (s-allele) transcript variant, or if they are equally likely to increase or decrease binding affinity. Figs 2A and 2B shows histograms of binding preferences for random and human RNA transcript fragments. For random transcripts, computer-generated indels near the middle of the fragment were used, while for human transcripts, naturally occurring indels from dbSNP [1] were analyzed. These distributions are symmetric and centered around zero, indicating that, on average, indels do not create a net preference for either the longer (l-allele) or shorter (s-allele) transcript variant.

While the average change in binding preference is close to 0, the histograms show a broad distribution, suggesting that individual indels can have substantial effects. To quantify the magnitude of these effects, we examined the standard deviation of the ΔΔG distributions. Figs 2C and 2D plot this standard deviation versus the distance from the indel for random and human transcript fragments. The shaded regions represent the 95% confidence intervals calculated via bootstrapping, providing statistical support for the observed trends.

The thermal energy at body temperature ($k_{B}T\approx 0.616\text{ kcal/mol}$) provides a useful benchmark for assessing these magnitudes; changes in binding energy smaller than *k*_*B*_*T* are less likely to overcome thermal noise and have consistent physiological consequences. In both Figs 2C and 2D, the standard deviation of ΔΔG clearly exceeds this thermal energy scale (dotted line), particularly for indels close to binding sites, suggesting that these structural effects can have physiologically relevant consequences. While *k*_*B*_*T* is a useful benchmark for energetic changes, the physiological effect of ΔΔG also depends on the specifics of the RBP-RNA interaction, like the binding regime. For instance, in a saturated system, even changes larger than *k*_*B*_*T* might not significantly alter RBP occupancy.

Fig 2 also reveals that the magnitude of the effect decreases as the distance between the indel and the binding site increases. This indicates that indels are most impactful when close to a binding site, and their influence diminishes with distance. While the data in Fig 2C appear to indicate that this effect further accelerates at a distance of around 40 nucleotides, we note that this is an artifact of the binding site approaching the end of the transcript fragment where there is a lower probability of base pairing; to verify this, S1 Fig confirms that this phenomenon is absent in longer transcripts.

### Larger indels have bigger effects on binding affinity.

**Fig 3:**
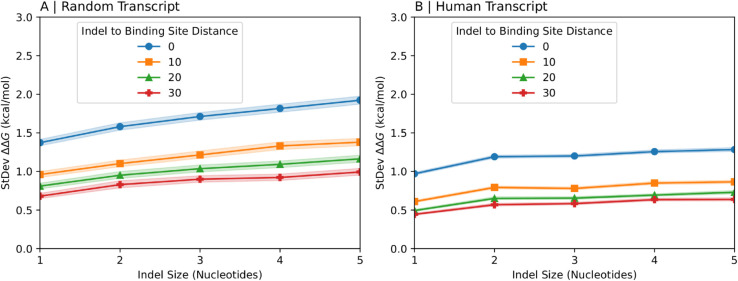
Dependence of binding preferences on indel size. The standard deviation of the binding preference calculated over many random (A) or human (B) transcript fragments of length 150nt near an indel are shown as a function of the size of the indel. The different lines correspond to different distances between the indel and the protein binding site on the transcript fragment (quoted in nucleotides). For human transcript fragments in (B) the indels are experimentally known indels from dbSNP; for random transcripts (A) the indels are generated by removing the desired number of nucleotides from the middle of the randomly generated transcript fragment. Shaded regions represent 95% confidence intervals.

We next examined how binding preference changes as a function of indel size. Fig 3 shows the standard deviation of the binding preference versus the size of the indel for synthetic indels in random transcript fragments (A) and natural indels in human transcript fragments (B). The standard deviation is greater for larger indels, indicating that larger indels are more likely to significantly influence protein binding affinity. However, the effect of increasing indel size on the rise in the standard deviation diminishes at larger indel sizes. This is perceptible here as a gradual decrease in the slope of the lines, but even more pronounced when the range of indel sizes is extended (S2 Fig). At large indel sizes, the rise in standard deviation of the binding preference in response to an increase in indel size appears to approach 0 (S2 Fig), indicating an upper limit for the extent to which an indel can affect protein binding affinity.

### Natural indels and natural binding sites are under selection to reduce impact on binding affinity

We observed striking differences between the effects of synthetic indels in random sequences and natural indels in human sequences. For example, the standard deviations in Fig 2C for random transcripts appear to be higher than those in Fig 2D for human transcript fragments. We hypothesize that this difference reflects purifying selection: indels that would significantly alter binding affinity at critical RNA-protein interaction sites are generally deleterious and thus are selected against, leading to their underrepresentation in the observed human population. In this section, we explore this hypothesis by examining changes in indel-induced binding preferences as we slowly transition from random transcript fragments with arbitrary binding sites to human transcript fragments with natural indels and binding sites. We note, that selection of indels in the general vicinity of functional sites has been studied before [8], but here we are rather looking at the question of selection due to the specific effect of the indel on RNA-protein binding affinity quantifiable by our approach.

#### Effect of randomly generated indels on binding affinity is similar between random and human transcript fragments.

So far, the term indel has been used slightly differently in human versus random transcript fragments. In random transcript fragments it referred to our arbitrary removal of nucleotides near the middle of the transcript fragment whereas in human transcript fragments, it referred to natural variations in the length of transcripts observed within the human populations as documented in dbSNP [1]. These naturally-observed variations represent a subset of indels where neither allele is deleterious enough to be eradicated from the population. In other words, observed variations are a selection on the space of possible indels for those whose size and location in the transcript make them less deleterious. Before investigating whether selection on *indel space* is influenced by binding preferences, we first assess whether selection on *sequence space* (the space of all possible arrangement of nucleotides) for human transcript fragments is influenced by binding preferences.

**Fig 4:**
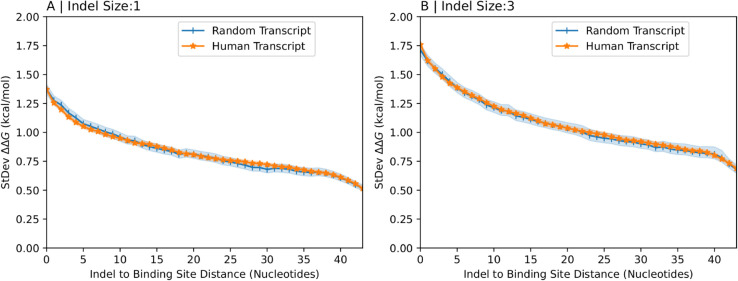
Binding preferences of synthetic indels in random vs human transcript fragments. The standard deviation of the binding preference calculated over many random (blue) and human (orange) transcript fragments of length 150nt near an indel are shown as a function of the distance between the indel and the protein binding site. For both transcript types the indels are generated by removing one (A) or three (B) nucleotides from the middle of the transcript fragment irrespective of the location of experimentally observed indels. Shaded regions represent 95% confidence intervals.

To assess this possibility, we compared binding preferences in random and human transcript fragments without reference to naturally occurring indels. The human transcript fragments were extracted randomly from human RNA sequences without requiring that they be close to a naturally occurring indel. We generated synthetic indels near the middle of the human transcript fragments identically to our approach for generating indels in random sequences by removing the desired number of nucleotides from the middle of the transcript fragment. Fig 4 shows the standard deviation of the binding preference versus the relative position of arbitrary binding sites for indels of size one (A) and three (B), respectively, with confidence intervals shown as shaded regions. In each panel, there are two curves, one corresponding to data for random transcript fragments and the other corresponding to data for human transcript fragments. It is evident that the standard deviation is approximately the same in random transcript fragments, the nucleotides of which are generated independently with frequencies of 1/4 each, and human transcript fragments. This suggests that the arrangement of nucleotides in arbitrary regions of natural human RNA transcript sequences including patterns driven by GC content or unusual dinucleotide frequencies does not make them more resistant to indel-induced binding preferences than they would be if the arrangement of nucleotides was uncorrelated with equal probability for the four bases.

#### Natural indels have a smaller effect on binding affinity than synthetic indels.

**Fig 5:**
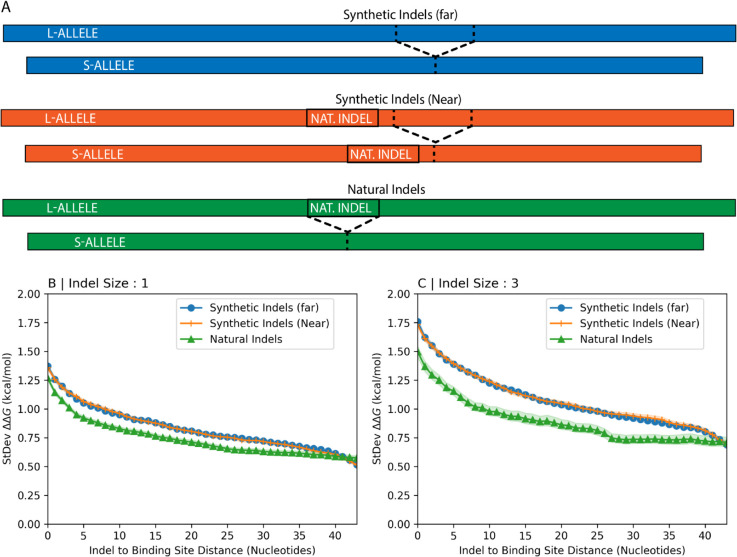
Comparison of binding preferences for natural and synthetic indels. Panel (A) illustrates three distinct strategies for generating indels in transcript fragments. The first strategy (Synthetic Indels - Far), involves choosing transcript fragments randomly from human transcripts without regard for the location of experimentally observed indels. In this approach, an indel is created by deleting a specified number of nucleotides near the center of the selected transcript fragment. The second strategy (Synthetic Indels - Near), involves selecting transcript fragments where a simulated indel is positioned close to an annotated indel location from dbSNP. With the final strategy (Natural Indels), the simulated indels are actual indels that are directly derived from dbSNP annotations. Panels (B) and (C) show the standard deviation of binding preference for each set of transcript fragments as a function of the distance between indels of size 1 nucleotide (Panel B) and 3 nucleotides (Panel C) and arbitrary 7-nucleotide binding sites located at increasing distances from it. Colors in Panels (B) and (C) correspond to the sets defined in Panel (A). Shaded regions represent 95% confidence intervals.

Now, we investigate the extent to which selection on the space of possible indel locations for naturally occurring indels is influenced by binding preferences. Our approach here involves generating synthetic indels that iteratively approach the location of natural indels and monitoring changes in binding preferences. We created 3 different sets of transcript fragments (Fig 5A). One set (Synthetic Indels - Far) contained no natural indels — synthetic indels were used to calculate binding preferences. In the second set (Synthetic Indels - Near), binding preferences were calculated using synthetic indels that were 25 nucleotides from natural indels. In the final set (Natural Indels), binding preferences were calculated using natural indels from dbSNP [1] located at the middle of the transcript fragment.

In Figs 5B and 5C, we show the standard deviation of binding preference as a function of the distance to the indel. The indel is fixed and we systematically calculate the effect on binding preferences at arbitrary 7-nucleotide binding sites located at increasing distances from it. The three curves in each panel are color-coded according to Fig 5A. We find that natural indels create smaller binding preferences than synthetic indels, while for synthetic indels it does not make a difference if they are close to natural indels or not. This is consistent with the hypothesis that naturally observed indels are under selection for reduced influence on protein binding affinity.

#### Proximity to natural protein binding sites reduces the effect of indels on binding affinity.

So far, binding preferences have been calculated using arbitrary binding sites – RNA regions for which we have no evidence of RNA-protein interactions. We may expect our results to differ at naturally-observed protein binding sites because protein-RNA interactions at natural binding site are more likely to be conserved. To explore this hypothesis, we repeated the analysis described in the previous section for transcripts proximal to experimentally-observed binding regions of the single-stranded RNA binding protein HuR. Given HuR’s established role in modulating the stability and translation of a wide array of mRNAs crucial for cellular homeostasis, including those involved in cell proliferation, stress responses, and oncogenesis [12], assessing selective pressures around its binding sites is particularly informative. Specifically, we selected transcript fragments that either contained an experimentally validated HuR binding site or were less than 200 nucleotides from such a site.

**Fig 6:**
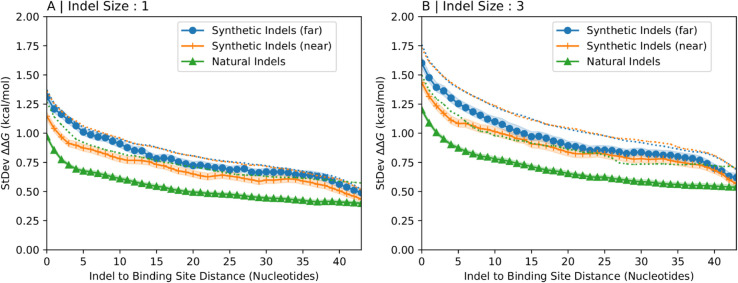
Binding preferences near HuR binding sites. The standard deviation of the binding preference calculated over many transcript fragments of length 150nt selected from the vicinity of experimentally validated HuR binding sites near an indel of length 1nt (A) and 3nt (B) are shown as a function of distance between the indel and the protein binding site. Each panel shows data for artificially generated indels far away from a natural indel in blue, artificially generated indels in the vicinity of a natural indel in orange, and natural indels from dbSNP in green (following the color code from Fig 5A). Shaded regions represent 95% confidence intervals. The dotted lines reproduce the data from Figs 5B and 5C for comparison, which were generated similarly but without specifically choosing transcript fragments near natural HuR binding sites.

Fig 6 shows the standard deviation of the binding preference versus the relative position of arbitrary binding sites color-coded according to the sets in Fig 5A. The dotted lines are reproduced from the curves in Figs 5B and 5C, respectively. Binding preferences calculated using transcript fragments proximal to natural binding sites (continuous lines) are lower than those for transcripts fragments extracted without regard for natural binding sites (dotted lines). This suggests that proximity to natural binding sites reduces binding preferences and conforms with our expectation that indels are less disruptive of binding affinity near natural binding sites. By comparing the effects of natural versus synthetic indels within these same HuR-proximal regions (Fig 6), our analysis inherently controls for any local sequence features, such as the U-richness of HuR binding motifs.

Additionally, Fig 6 suggests that proximity to natural binding sites accentuates differences between synthetic indels – synthetic indels that are far from natural indels have a larger effect on binding affinity than synthetic indels closer to natural indels only when transcript fragments are proximal to binding sites. This implies that in the vicinity of natural protein binding sites, mutations near natural indels tend to have a diminished effect on binding affinity than mutations farther from natural indels.

#### Effect of indels on binding affinity is significantly smaller at natural protein binding sites than at synthetic protein binding sites.

We have established that proximity to natural binding sites reduces the effect of indels on binding affinity. We take this idea one step further and compare binding preferences calculated using natural binding sites with those calculated using synthetic binding sites. In prior sections, even in the cases where transcript fragments were extracted to be proximal to HuR binding regions, a selection of 7mers on the transcript fragments were arbitrarily assigned to act as binding sites. Here, we determined the 7mer that is most likely to serve as the true HuR binding site *in vivo*. An investigation by Mukerjee *et al.* resolved the location of HuR binding sites to a set of approximately 25-nucleotide loci [21]. The RBPBind software [6], in conjuction with an RNAcompete dataset detailing the relative preferences of HuR for each 7mer [22], was used to determine the likely 7-nucleotide binding site of HuR within each of the experimentally determined 25-nucleotide loci. Binding preferences were then calculated using these natural indels and the identified natural HuR binding sites, which could be located on either the 5’ or 3’ side of the indel within the transcript fragment. For comparison, control regions were generated by taking these same ∼25-nucleotide HuR binding loci from Mukherjee *et al*. and shifting their coordinates 300 nucleotides upstream (towards the 5’ end) on their respective transcripts. The identical RBPBind and RNAcompete-based procedure just described was then applied to these ’shifted control regions’ to identify the most probable 7-nucleotide synthetic binding site within each. Binding preferences for these synthetic sites were subsequently calculated in the same manner.

**Fig 7:**
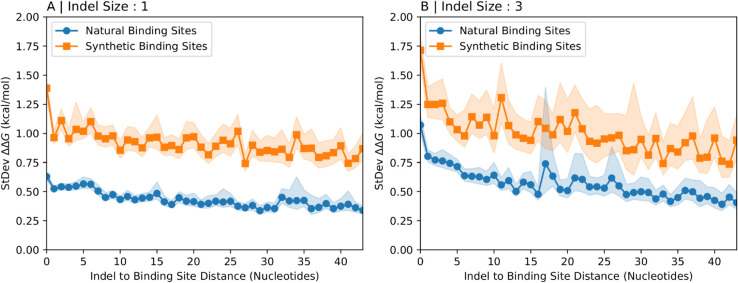
Binding preferences for natural indels at authentic vs synthetic HuR binding sites. The standard deviation of the binding preference calculated over many transcript fragments of length 150nt near an indel annotated in dbSNP of length 1nt (A) and 3nt (B) are shown as a function of distance between the indel and the protein binding site. Each panel shows data for synthetic protein binding sites in orange and for experimentally validated HuR protein binding sites in blue. Shaded regions represent 95% confidence intervals.

Fig 7 shows the standard deviation of the binding preferences versus the relative position of binding sites for indels annotated in dbSNP of size one and three. Binding preferences for the orange curve were calculated using synthetic binding sites and binding preferences for the blue curve were calculated using HuR binding sites. It is clear that the standard deviation is significantly smaller when HuR binding sites are used to calculate binding preferences. This provides additional evidence that purifying selection acts to preserve HuR binding function by removing indels that would significantly disrupt binding affinity.

## Discussion

The sequence of an RNA molecule dictates not only where a protein can bind but also the structural landscape that makes a binding site accessible. We have shown that indels can alter this landscape from a distance, creating significant changes in RBP binding affinity for the two alleles. Overall, proteins show no systematic preference for either allele (Figs 2A and 2B). However, indel mutations, especially those large enough or positioned near enough binding sites, often create binding preferences that exceed the thermal energy scale (Fig 2C,D). This suggests that an indel outside an RNA molecule’s binding site can influence the folding and base-pairing patterns within the binding site, leading to different affinities of RNA-binding proteins for the two alleles. When the difference in affinity surpasses the thermal energy threshold, it suggests potential physiological impact.

While the low rate of indel mutations at any single locus makes it difficult to infer selection, our approach gains statistical power by aggregating the predicted effect sizes (ΔΔG) across thousands of distinct indel–HuR-binding-site pairs. This design allows us to detect the signature of selection acting on the magnitude of an indel’s effect, a signal that would be difficult to distinguish from the random fluctuations of genetic drift at any individual locus. We further show that binding preferences are subject to natural selection and thus likely physiologically relevant. Specifically, indel polymorphisms create smaller binding preferences when near HuR binding sites (Fig 6). This suggests that the action of natural selection over time has resulted in the preferential persistence of HuR binding sites located within sequence contexts that are resilient to nearby indels. HuR regulates numerous transcripts essential for key cellular functions, including the control of cell cycle, apoptosis, and inflammatory responses [9,12]. Disruptions to HuR’s regulatory network by indels could therefore have widespread deleterious effects, explaining the selective pressure to preserve the integrity of these interactions. The reduced impact of natural versus synthetic indels suggests that indel mutations with significant effects on binding affinity are deleterious and thus purged from the population by purifying selection.

An intriguing alternative is that a biased mutational process, rather than selection, could contribute to the patterns we observe. It is well-established that indel formation is non-random, with mechanisms like polymerase slippage creating low complexity mutations in repetitive regions [8,23]. The possibility that these mechanisms might also be inherently biased towards generating structurally innocuous indels could explain the baseline difference we observe between natural and synthetic indels. However, a key prediction of the purifying selection hypothesis is that this effect should be strongest in functionally important regions, a prediction that Fig 6 allows us to test directly. As shown in the figure, the difference in structural impact between natural indels (solid green line) and synthetic indels (solid blue line) is visibly larger in the context of HuR binding sites compared to the same difference observed in average transcript regions (the corresponding dotted lines, reproduced from Fig 5). While a general mutational bias is unlikely to be sensitive to proximity to a functional element, the strength of purifying selection is expected to be greatest where mutations have the largest functional cost. This context-dependent amplification of the signal strongly suggests that while a generative bias may exist, it acts in concert with purifying selection to shape the landscape of observed indel effects.

This work provides strong evidence that indels can disrupt physiologically significant protein-RNA interactions from a distance, with such effects being constrained by purifying selection. While this may be a general principle for many RNA-binding proteins, we have demonstrated its action for the specific case of HuR. To facilitate further investigation into this model system, we provide a supplementary table (S1 Table) cataloging loci where natural indels are predicted to significantly impact HuR binding affinity. The table is filtered to include only variants where the predicted binding preference (|ΔΔG|) exceeds the thermal energy (*k*_*B*_*T*). These variants are prime candidates for the crucial next step of establishing statistically rigorous links to disease, for instance through genome-wide association studies (GWAS). For those variants that show a robust disease association, moving from correlation to mechanistic insight will then require algorithms that can model the full structure of the RNA-protein complex, such as AlphaFold [24], and, crucially, direct experimental validation.

## Materials and methods

### Creating random transcript fragments.

Averages and standard deviations of binding preferences were calculated over 4000 RNA fragments, each 150 nucleotides long. In the case of random fragments, the probability of selecting any of the four nucleotides (Adenine, Cytosine, Guanine, and Uracil) was equal at every position. To assess the robustness of our results across different fragment lengths, we compared binding predictions between 150-nucleotide and 250-nucleotide transcripts (see S1 Fig and S2 Fig).

### Generating synthetic indels and binding sites in random transcript fragments.

Much of our analysis relies on artificially generated indels, rather than those documented in the dbSNP database. Our approach to creating these indels is illustrated in Fig 1. Specifically, nucleotides were deleted from one end of a 50-nucleotide window located at the center of each 150-nucleotide transcript fragment. For each fragment, we generated five different short-alleles (s-alleles), with 1, 2, 3, 4, or 5 nucleotides removed from the corresponding long-allele (l-allele), respectively.

Arbitrary binding sites were simulated within the 50-nucleotide region located 3’ (downstream) to these indels. For each binding site, the binding preference (ΔΔG) was calculated as the difference in binding affinity between the l-allele and the s-allele (see below). This calculation was repeated for all possible 7-nucleotide binding sites within the 50-nucleotide binding region. Importantly, this approach maintains a consistent distance between the indel and each binding site across all five alleles, as depicted in Fig 1. We recognized that placing binding sites near the ends of these fragments could introduce boundary effects on secondary structure prediction. We therefore explicitly test for these ’end-effects’ and demonstrate using longer transcripts that they do not affect our overall conclusions (see discussion of Figs 2C and S1).

While the analysis described here places the arbitrary binding site always 3’ (downstream) of the indel, we repeated the analysis for one of the data sets with arbitrary binding sites placed 5’ (upstream) of the indel. As S3 Fig demonstrates, the results are nearly identical, confirming that our findings are not dependent on the relative orientation of the indel and binding site.

### Generating synthetic indels and binding sites in human transcript fragments.

We retrieved all human primary RNA transcripts from the Ensembl database (release 100) [25]. From these transcripts, we extracted non-overlapping 150-nucleotide transcript fragments, which served as our l-allele sequences. To create short-allele (s-allele) sequences, we deleted a stretch of 1–5 nucleotides from each fragment following the approach illustrated in Fig 1. Arbitrary binding sites were simulated at all possible 7-mers within the 50-nucleotide region downstream to the indel.

### Obtaining human indels.

Human indel specifications were obtained in VCF file format from the Ensembl database (release 100) [25]. The VCF files included various types of genomic variations; therefore, indels were extracted using VCFtools with the --keep-only-indels option [26]. The filtered VCF file contained entries for each human indel, including its alleles and genomic coordinates, based on the GRCh38 genome assembly.

### Selecting human indels and arbitrary binding sites in human transcript fragments.

Using indel coordinates, we identified transcripts containing indels from the primary RNA transcript dataset obtained from the Ensembl database (release 100) [25], restricting the analysis to indels shorter than six nucleotides. Transcript fragment boundaries were chosen such that the indels were positioned at the center of each 150-nucleotide transcript fragment. The longer alleles (l-alleles) were derived from the longer of the two naturally occurring alleles, while shorter alleles (s-alleles) were generated using two approaches. (1) The s-alleles labeled “Synthetic Indels - Near” in Fig 5 were created with the protocol illustrated in Fig 1, simulating synthetic indels approximately 20 nucleotides from the documented indel at the center of the transcript fragment. Arbitrary binding sites were simulated at all possible 7-mers within the final 50-nucleotide region of the transcript fragment, as shown in Fig 1. (2) The s-alleles labeled “Natural Indels” in Fig 5 were generated by using the documented indel at the center of the transcript fragment to create the shorter allele observed in the human population. In this case, the arbitrary binding sites were chosen at all possible 7-mers within the 50-nucleotide region immediately following the indel that ends approximately 20 nucleotides from the end of the transcript fragment.

### Obtaining HuR binding regions.

We retrieved HuR binding regions previously identified by Mukherjee et al. from the doRiNA database [21,27]. These entries define approximately 25 nucleotide long binding regions that encompass the specific 7 nucleotide binding site of the HuR protein. Since the original coordinates were based on the hg19 genome assembly, we used UCSC’s LiftOver tool (http://genome.ucsc.edu/cgi-bin/hgLiftOver) [28] to convert these coordinates to their equivalent positions in the GRCh38 assembly.

### Obtaining human transcript fragments with human indels and HuR binding regions.

From the data set of human primary RNA transcripts, we extracted 150-nucleotide sequences containing both a documented human indel and a complete HuR binding region. The boundaries of the fragments were chosen such that the indels were positioned at the center of the extracted fragments. The l-allele was obtained using the allele specification with the longer sequence and the appropriate nucleotides were deleted to obtain the s-allele.

### Finding HuR binding sites within HuR binding regions.

The experimentally determined 25-nucleotide HuR binding regions are expected to contain the 7-nucleotide HuR binding sites [21]. To pinpoint the location of the HuR binding site within these regions, we used the RBPBind software [6].

RBPBind includes a function that calculates the probability of HuR binding to every 7mer in an RNA sequence. This function takes three inputs: the relative binding preferences of HuR for all possible 7-nucleotide sequences, the RNA sequence, and the concentration of HuR near the RNA molecule. The relative binding preferences of HuR for all 7-nucleotide sequences was obtained from an RNACompete dataset [22]. The concentration parameter was set to the effective dissociation constant between HuR and the input RNA sequence, as computed by RBPBind using the RNACompete dataset.

For each pair of alleles (l-allele and s-allele), we added the binding probabilities of the two alleles for all 7mers shared between them. Any 7mers in the l-allele that were partially or completely absent from the s-allele were excluded from this calculation. The binding site of each pair of alleles was defined as the 7mer within the binding region that had the maximum summed probability.

### Computing binding preferences.

The Vienna package was used to compute the binding preference (ΔΔG), which quantifies the difference in binding affinity between the l-allele and the s-allele at the same binding site [17].

For each fragment, the change in free energy associated with protein binding was approximated as $\Delta G=G_{\text{constraint}}\;-\;G_{\text{none}}$. Here, $G_{\text{constraint}}$ represents the free energy of folding with base-pairing constraints applied at the binding site, while $G_{\text{none}}$ represents the free energy of folding without these constraints. Introducing base-pairing constraints increases the free energy of folding (makes it less negative), resulting in ΔG being positive.

To evaluate the contribution of the indel to the free energy of protein binding, we calculated $\Delta \Delta G=\Delta G_{\text{l}}\;-\;\Delta G_{\text{s}}$. This is the difference between ΔG for the l-allele and ΔG for the s-allele at the same binding site. A positive ΔΔG indicates that protein binding to the l-allele results in a greater increase in the free energy of folding compared to the s-allele, suggesting a stronger binding preference for the s-allele. Conversely, a negative ΔΔG implies a stronger protein binding affinity for the l-allele.

### Statistical analysis and sample sizes.

Confidence intervals for the standard deviation of binding preferences (ΔΔG) were calculated using a bootstrap method with 5,000 resamples. For analyses involving synthetic indels in random transcript fragments, a set of 4,000 sequences was used. For synthetic indels in arbitrary human transcript fragments, a set of 20,000 sequences was used. For synthetic indels in human transcript fragments close to HuR binding sites, a set of 5,750 sequences was used. For analyses involving human transcript fragments and natural indels, the number of available sequences is intrinsically variable as it depends on the frequency of each indel size in the dbSNP database. This variability in sample size is naturally reflected in the width of the corresponding confidence intervals, with larger samples generally resulting in tighter confidence bounds on our estimates. To ensure computational feasibility while maintaining robust statistics, datasets with more than 20,000 available sequences associated with the same indel size and binding site location were randomly subsampled to N=20,000. Specifically, for natural indels irrespective of HuR binding sites, 20,000, 6,567, 4,275, 3,696, and 1,394 sequences were used for indels of length 1, 2, 3, 4, and 5, respectively. For natural indels in the vicinity of HuR binding sites 20,000 sequences were used for indel lengths of 1 through 4, and 14,194 sequences were used for indel length 5. Lastly, in Fig 7 a total of 161,845 indels of length 1 at synthetic binding sites, 205,096 indels of length 1 near natural HuR binding sites, 40,550 indels of length 3 at synthetic binding sites, and 55,610 indels of length 3 near natural HuR binding sites were used; these are distributed across the different distances between the indel and the synthetic or natural binding site depending on the detected locations of the respective HuR binding sites and the variability of the confidence intervals represents the variability of the number of sequences at every given distance.

## Supporting information

S1 TableCandidate indels with predicted functional impact on HuR binding.This table lists natural human indels predicted to have a physiologically significant impact ($|\Delta \Delta G|>k_{B}T$) on the binding affinity of the HuR protein. The columns are as follows: **chromosome**, the chromosome on which the indel is located; **indel_genomic_coordinate**, the genomic coordinate (GRCh38) of the first nucleotide of the indel on the reference allele; **dbSNP_ID**, the reference SNP ID for the indel from the dbSNP database; **ref_allele**, the reference allele sequence; **alt_allele**, the alternate allele sequence; **binding_site_locus**, the genomic coordinates of the computationally determined 7-nucleotide HuR binding site; **binding_region_locus**, the genomic coordinates of the experimentally determined 25-nucleotide region from which the binding site was identified; **delta_delta_G**, the predicted change in binding preference in kcal/mol, where a positive value indicates stronger binding to the shorter allele; **transcript_ids**, a semicolon-separated list of Ensembl transcript IDs that overlap the indel’s location.(XLS)

S1 Fig Standard deviation of the binding preference for RNA binding proteins for indels on random transcripts of different lengths. The standard deviation of the binding preference calculated over many random 150-nucleotide (blue) and 250-nucleotide (orange) transcript fragments are shown as a function of the distance between the indel and the protein binding site. For both transcript types, the indels are generated by removing one (A) or three (B) nucleotides from the middle of the transcript fragment. Shaded regions represent 95% confidence intervals.
(TIFF)

S2 Fig Dependence of standard deviation of the binding preference for RNA binding proteins on indel size in random transcripts of different lengths. The standard deviation of the binding preference calculated over many random 150-nucleotide (blue) and 250-nucleotide (orange) transcript fragments are shown as a function of indel size. In panel (A), the protein binding site is positioned right next to the indel whereas in panel (B) this distance is 10 nucleotides. Shaded regions represent 95% confidence intervals.
(TIFF)

S3 Fig Effect of binding site orientation relative to natural indels. The standard deviation of the binding preference for natural indels near HuR binding sites, as a function of the distance between the indel and a 7-nucleotide binding site. This figure reproduces the ’Natural Indels’ data from Fig 6 (blue line), where binding sites were simulated in the 3’ region downstream of the indel. The orange line shows the result of a parallel analysis where binding sites were simulated in the 5’ region upstream of the indel. The analysis is shown for indels of size 1 (A) and size 3 (B). The near-identical results demonstrate that the orientation of the binding site relative to the indel does not significantly influence the magnitude of the effect on binding affinity. Shaded regions represent 95% confidence intervals.
(TIFF)

## References

[1] Sherry ST, Ward M, Sirotkin K. dbSNP-database for single nucleotide polymorphisms and other classes of minor genetic variation. Genome Res. 1999;9(8):677–9. doi: 10.1101/gr.9.8.677 10447503

[2] Mayya VK, Duchaine TF. Ciphers and executioners: How 3’-untranslated regions determine the fate of messenger RNAs. Front Genet. 2019;10:6. doi: 10.3389/fgene.2019.00006 30740123 PMC6357968

[3] Gerstberger S, Hafner M, Tuschl T. A census of human RNA-binding proteins. Nat Rev Genet. 2014;15(12):829–45. doi: 10.1038/nrg3813 25365966 PMC11148870

[4] Hackermüller J, Meisner N-C, Auer M, Jaritz M, Stadler PF. The effect of RNA secondary structures on RNA-ligand binding and the modifier RNA mechanism: A quantitative model. Gene. 2005;345(1):3–12. doi: 10.1016/j.gene.2004.11.043 15716109

[5] Lin Y-H, Bundschuh R. RNA structure generates natural cooperativity between single-stranded RNA binding proteins targeting 5’ and 3’UTRs. Nucleic Acids Res. 2015;43(2):1160–9. doi: 10.1093/nar/gku1320 25550422 PMC4333377

[6] Gaither J, Lin Y-H, Bundschuh R. RBPBind: Quantitative prediction of protein-RNA interactions. J Mol Biol. 2022;434(11):167515. doi: 10.1016/j.jmb.2022.167515 35662470

[7] Halvorsen M, Martin JS, Broadaway S, Laederach A. Disease-associated mutations that alter the RNA structural ensemble. PLoS Genet. 2010;6(8):e1001074. doi: 10.1371/journal.pgen.1001074 20808897 PMC2924325

[8] Montgomery SB, Goode DL, Kvikstad E, Albers CA, Zhang ZD, Mu XJ, et al. The origin, evolution, and functional impact of short insertion-deletion variants identified in 179 human genomes. Genome Res. 2013;23(5):749–61. doi: 10.1101/gr.148718.112 23478400 PMC3638132

[9] Brennan CM, Steitz JA. HuR and mRNA stability. Cell Mol Life Sci. 2001;58(2):266–77. doi: 10.1007/PL00000854 11289308 PMC11146503

[10] López de Silanes I, Zhan M, Lal A, Yang X, Gorospe M. Identification of a target RNA motif for RNA-binding protein HuR. Proc Natl Acad Sci U S A. 2004;101(9):2987–92. doi: 10.1073/pnas.0306453101 14981256 PMC365732

[11] Fan XC, Steitz JA. Overexpression of HuR, a nuclear-cytoplasmic shuttling protein, increases the in vivo stability of ARE-containing mRNAs. EMBO J. 1998;17(12):3448–60. doi: 10.1093/emboj/17.12.3448 9628880 PMC1170681

[12] Wang J, Guo Y, Chu H, Guan Y, Bi J, Wang B. Multiple functions of the RNA-binding protein HuR in cancer progression, treatment responses and prognosis. Int J Mol Sci. 2013;14(5):10015–41. doi: 10.3390/ijms140510015 23665903 PMC3676826

[13] Ripin N, Boudet J, Duszczyk MM, Hinniger A, Faller M, Krepl M, et al. Molecular basis for AU-rich element recognition and dimerization by the HuR C-terminal RRM. Proc Natl Acad Sci U S A. 2019;116(8):2935–44. doi: 10.1073/pnas.1808696116 30718402 PMC6386705

[14] D’Agostino VG, Adami V, Provenzani A. A novel high throughput biochemical assay to evaluate the HuR protein-RNA complex formation. PLoS One. 2013;8(8):e72426. doi: 10.1371/journal.pone.0072426 23951323 PMC3741180

[15] Shatoff E, Bundschuh R. Single nucleotide polymorphisms affect RNA-protein interactions at a distance through modulation of RNA secondary structures. PLoS Comput Biol. 2020;16(5):e1007852. doi: 10.1371/journal.pcbi.1007852 32379750 PMC7237046

[16] Forties RA, Bundschuh R. Modeling the interplay of single-stranded binding proteins and nucleic acid secondary structure. Bioinformatics. 2010;26(1):61–7. doi: 10.1093/bioinformatics/btp627 19889798

[17] Lorenz R, Bernhart SH, Höner Zu Siederdissen C, Tafer H, Flamm C, Stadler PF, et al. ViennaRNA package 2.0. Algor Mol Biol. 2011;6(1):26. doi: 10.1186/1748-7188-6-26 22115189 PMC3319429

[18] Mathews DH, Disney MD, Childs JL, Schroeder SJ, Zuker M, Turner DH. Incorporating chemical modification constraints into a dynamic programming algorithm for prediction of RNA secondary structure. Proc Natl Acad Sci U S A. 2004;101(19):7287–92. doi: 10.1073/pnas.0401799101 15123812 PMC409911

[19] McCaskill JS. The equilibrium partition function and base pair binding probabilities for RNA secondary structure. Biopolymers. 1990;29(6–7):1105–19. doi: 10.1002/bip.360290621 1695107

[20] Layton DM, Bundschuh R. A statistical analysis of RNA folding algorithms through thermodynamic parameter perturbation. Nucleic Acids Res. 2005;33(2):519–24. doi: 10.1093/nar/gkh983 15673712 PMC548333

[21] Mukherjee N, Corcoran DL, Nusbaum JD, Reid DW, Georgiev S, Hafner M, et al. Integrative regulatory mapping indicates that the RNA-binding protein HuR couples pre-mRNA processing and mRNA stability. Mol Cell. 2011;43(3):327–39. doi: 10.1016/j.molcel.2011.06.007 21723170 PMC3220597

[22] Ray D, Kazan H, Chan ET, Peña Castillo L, Chaudhry S, Talukder S, et al. Rapid and systematic analysis of the RNA recognition specificities of RNA-binding proteins. Nat Biotechnol. 2009;27(7):667–70. doi: 10.1038/nbt.1550 19561594

[23] Garcia-Diaz M, Kunkel TA. Mechanism of a genetic glissando: Structural biology of indel mutations. Trends Biochem Sci. 2006;31(4):206–14. doi: 10.1016/j.tibs.2006.02.004 16545956

[24] Abramson J, Adler J, Dunger J, Evans R, Green T, Pritzel A, et al. Accurate structure prediction of biomolecular interactions with AlphaFold 3. Nature. 2024;630(8016):493–500. doi: 10.1038/s41586-024-07487-w 38718835 PMC11168924

[25] Yates AD, Achuthan P, Akanni W, Allen J, Allen J, Alvarez-Jarreta J, et al. Ensembl 2020. Nucleic Acids Res. 2020;48(D1):D682–D688. doi: 10.1093/nar/gkz966 31691826 PMC7145704

[26] Danecek P, Auton A, Abecasis G, Albers CA, Banks E, DePristo MA, et al. The variant call format and VCFtools. Bioinformatics. 2011;27(15):2156–8. doi: 10.1093/bioinformatics/btr330 21653522 PMC3137218

[27] Blin K, Dieterich C, Wurmus R, Rajewsky N, Landthaler M, Akalin A. DoRiNA 2.0—Upgrading the doRiNA database of RNA interactions in post-transcriptional regulation. Nucleic Acids Res. 2015;43(Database issue):D160-7. doi: 10.1093/nar/gku1180 25416797 PMC4383974

[28] Kent WJ, Sugnet CW, Furey TS, Roskin KM, Pringle TH, Zahler AM, et al. The human genome browser at UCSC. Genome Res. 2002;12(6):996–1006. doi: 10.1101/gr.229102 12045153 PMC186604

