# Supplementary figures and images for: The structural effects of indel polymorphisms outside the binding site on RNA-protein interactions are shaped by selection
Source: PLoS Comput Biol. 2025 Oct 21;21(10):e1013604. doi: 10.1371/journal.pcbi.1013604 (PMC12551953; doi:10.1371/journal.pcbi.1013604)

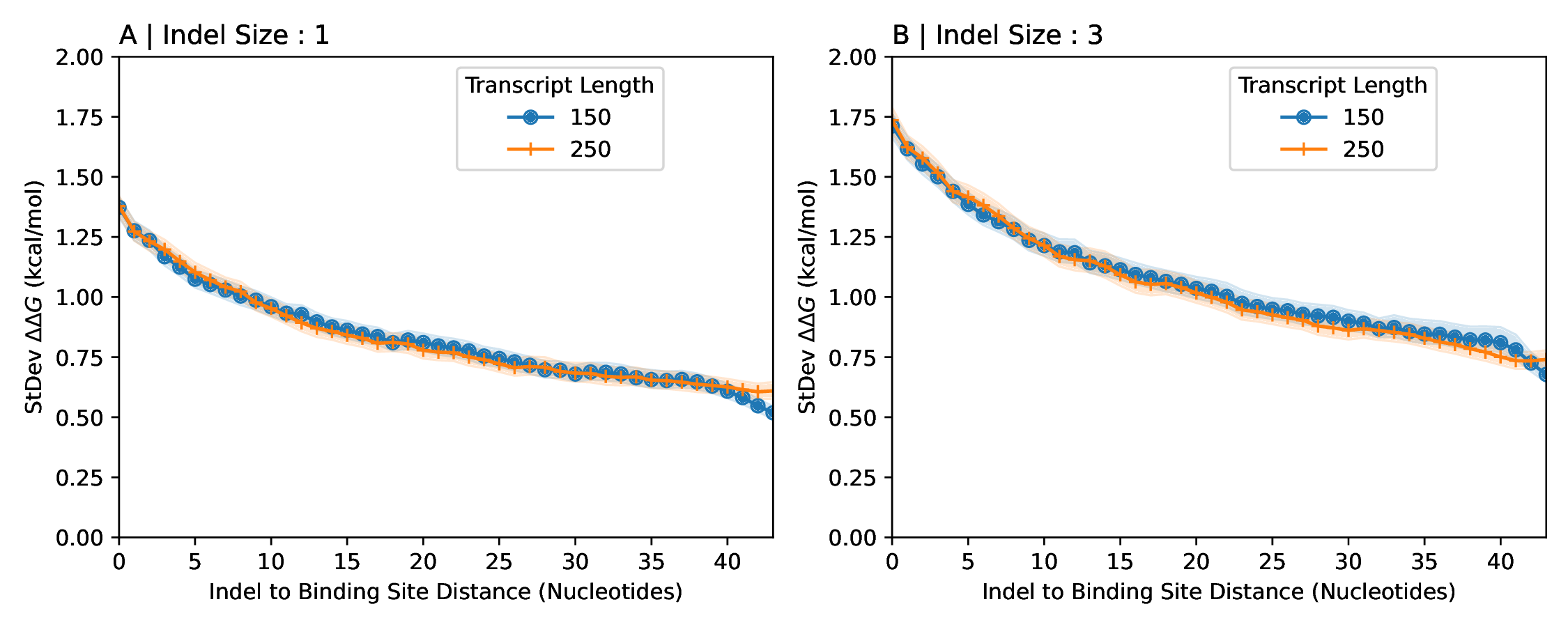

Supplement: S1 Fig — The standard deviation of the binding preference calculated over many random 150-nucleotide (blue) and 250-nucleotide (orange) transcript fragments are shown as a function of the distance between the indel and the protein binding site. For both transcript types, the indels are generated by removing one (A) or three (B) nucleotides from the middle of the transcript fragment. Shaded regions represent 95% confidence intervals. (TIFF) [file pcbi.1013604.s002.tif]

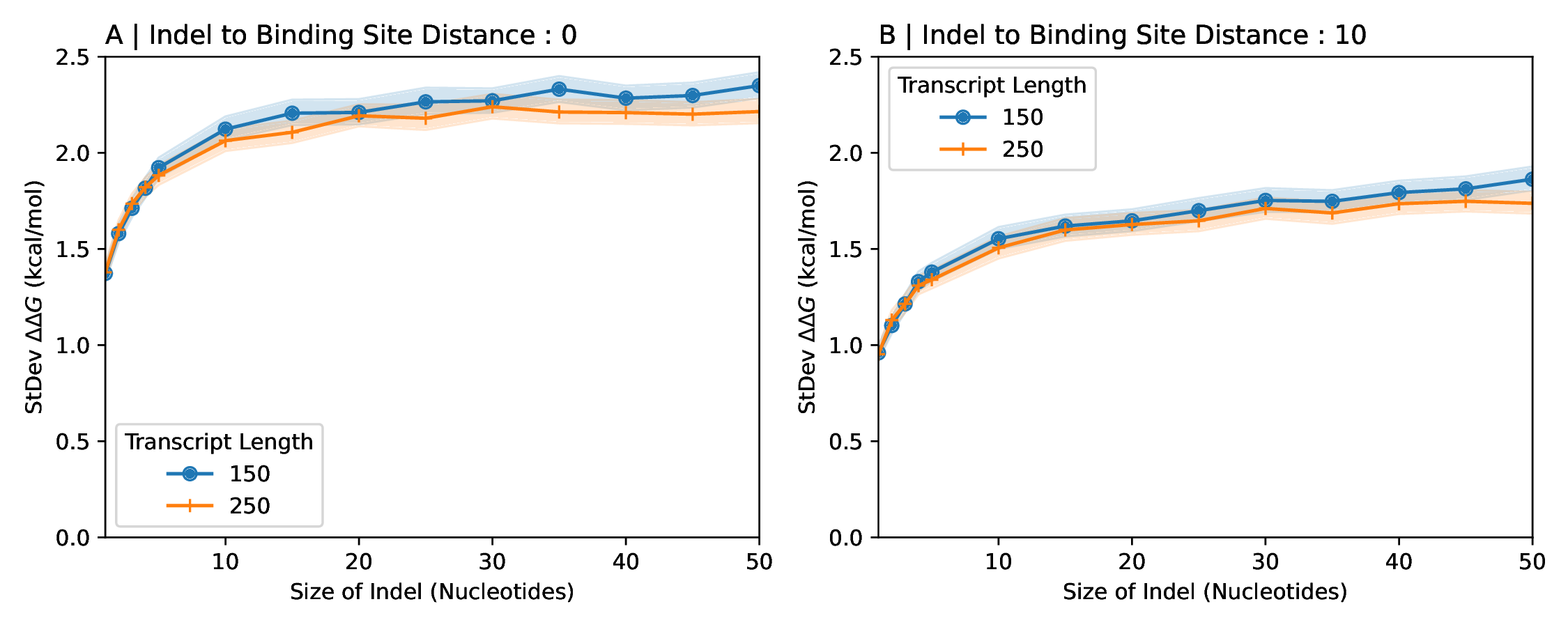

Supplement: S2 Fig — The standard deviation of the binding preference calculated over many random 150-nucleotide (blue) and 250-nucleotide (orange) transcript fragments are shown as a function of indel size. In panel (A), the protein binding site is positioned right next to the indel whereas in panel (B) this distance is 10 nucleotides. Shaded regions represent 95% confidence intervals. (TIFF) [file pcbi.1013604.s003.tif]

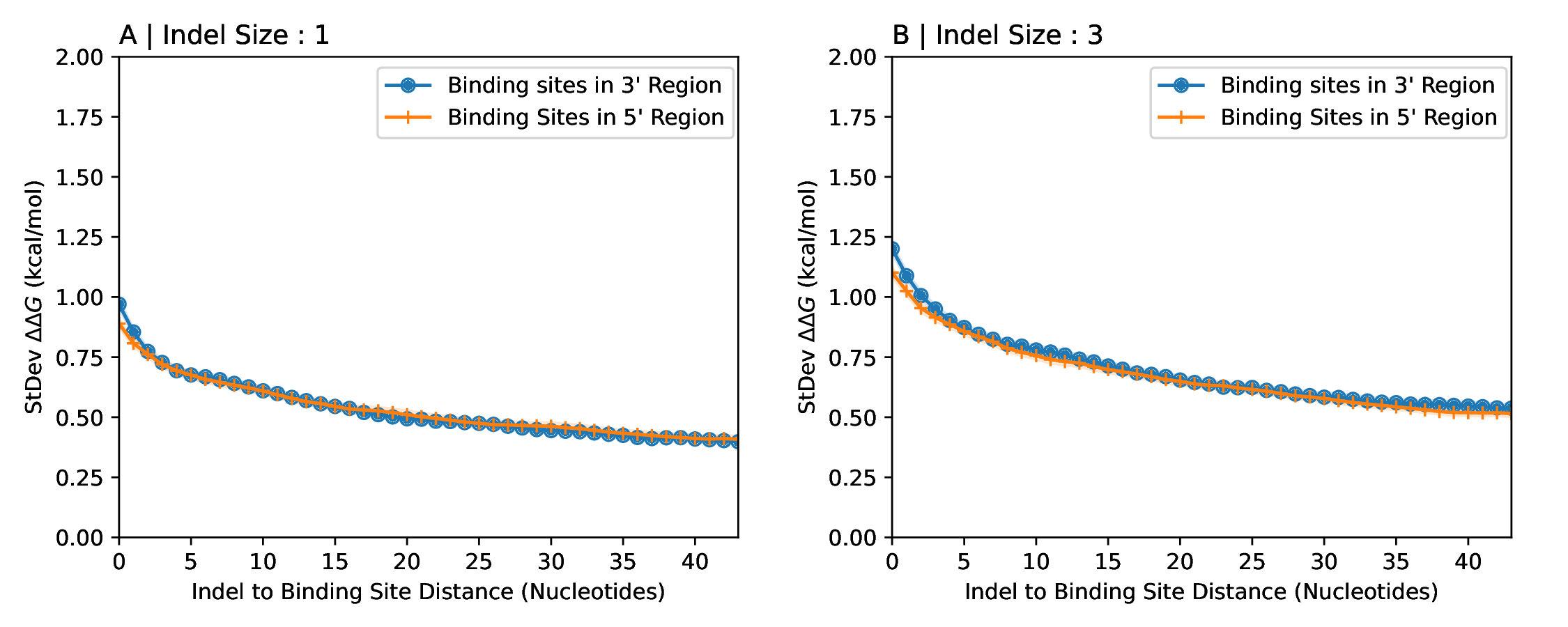

Supplement: S3 Fig — The standard deviation of the binding preference for natural indels near HuR binding sites, as a function of the distance between the indel and a 7-nucleotide binding site. This figure reproduces the ’Natural Indels’ data from Fig 6 (blue line), where binding sites were simulated in the 3’ region downstream of the indel. The orange line shows the result of a parallel analysis where binding sites were simulated in the 5’ region upstream of the indel. The analysis is shown for indels of size 1 (A) and size 3 (B). The near-identical results demonstrate that the orientation of the binding site relative to the indel does not significantly influence the magnitude of the effect on binding affinity. Shaded regions represent 95% confidence intervals. (TIFF) [file pcbi.1013604.s004.tif]
